# Antidepressant-like and neuroprotective effects of pine needle extracts: evidence from behavioral, transcriptomic, and biochemical studies

**DOI:** 10.17179/excli2025-8720

**Published:** 2025-10-17

**Authors:** Hisako Iwahashi Ogawa, Eiji Yasaka, Shinji Kondo, Farhana Ferdousi, Mitsutoshi Nakajima, Hiroko Isoda

**Affiliations:** 1 Degree Programs in Life and Earth Sciences, Graduate School of Science and Technology, University of Tsukuba, Tsukuba, Ibaraki, 305-8572, Japan; 2 Arakawa Chemical Industries, Ltd.,1-3-7, Hiranomachi, Chuo-ku, Osaka,541-0046, Japan; 3 Alliance for Research on the Mediterranean and North Africa (ARENA), University of Tsukuba, Tsukuba, Ibaraki 305-8572 Japan; 4 Institute of Life and Environmental Sciences, University of Tsukuba, Tsukuba, Ibaraki, 305-8575, Japan; 5 Open Innovation Laboratory for Food and Medicinal Resource Engineering (FoodMed-OIL), National Institute of Advanced Industrial Science and Technology (AIST), Tsukuba, Ibaraki, 305-0821, Japan; 6 MED R&D Co. Ltd., Tsukuba, Ibaraki, 305-8572, Japan

**Keywords:** depression, transcriptomics, neuroinflammation, tail suspension test, neuroprotection, apelin

## Abstract

Neuroinflammation is a key characteristic associated with neurological disorders, particularly depression and anxiety. This study aims to evaluate the neuroprotective and antidepressant-like effects of pine needle (PN) extracts in an LPS-induced neuroinflammation mouse model. Following seven days of oral administration of PN, the tail suspension test demonstrated a significant reduction in immobility time in PN-treated mice compared to LPS controls, surpassing the effect of the standard antidepressant bupropion. To elucidate the underlying mechanisms, we conducted a whole-genome microarray analysis. This analysis highlighted pathways related to neuroprotection, synaptic plasticity, and pro-inflammatory cytokine regulation, with a notable enrichment in the Apelin signaling pathway. Quantitative PCR analysis revealed that PN treatment increased the levels of Apelin and its receptor while decreasing proinflammatory cytokines *Tnfa* and *IL1b* in the hippocampus. ELISA further demonstrated elevated levels of key neurotransmitters, including dopamine and noradrenaline, in the mouse hippocampus. Additionally, we performed GC/MS analysis to identify bioactive compounds in PN, revealing D-Pinitol and Shikimic acid as major constituents. Importantly, catechol exhibited significant neuroprotective effects, and similar protective effects were also noted in the mixed compositions. The MTT assay showed that PN and its compounds significantly improved cell metabolic activity against dexamethasone-induced cytotoxicity. In conclusion, our findings highlight the potential of PN as a natural therapeutic agent for depressive symptoms, promoting neuroprotection, enhancing neurotransmitter levels, and modulating inflammatory responses.

See also the graphical abstract(Fig. 1).

## Abbreviations

5-HT: serotonin

APJ: apelin receptor

APLN: apelin

APLNR: apelin receptor

BDNF: brain-derived neurotrophic factor

DA: dopamine

DEX: dexamethasone

GC/MS: gas chromatography-mass spectrometry

HPLC: high performance liquid chromatography

IL-1β: interleukin 1 beta

KEGG: Kyoto Encyclopedia of Genes and Genomes

LPS: lipopolysaccharide

MTT: 3-(4,5-di-methylthiazol-2-yl)-2,5-diphenyltetrazolium bromide, yellow tetrazole

NA: norepinephrine

NTRKs: neurotrophic tyrosine kinases

PN: pine needle

TNF: tumor necrosis factor

TRKA: tropomyosin receptor kinase A

TRKB: tropomyosin receptor kinase B

TST: tail suspension test

## Introduction

Depression is a ubiquitous mood disorder that poses significant challenges to mental health globally, with an estimated 360 million individuals affected by this condition (Institute of Health Metrics and Evaluation. Global Health Data Exchange (GHDx). https://vizhub.healthdata.org/gbd-results/ (Accessed 22 January 2025)). Depression is a multifactorial disorder characterized by complex pathophysiological mechanisms, including neuroinflammation, synaptic dysfunction, alterations in neurotransmitter systems, and impaired neurotrophic signaling. Despite advancements in treatment modalities, there exists an ongoing imperative to discover novel, effective, and safer therapeutic options that possess antidepressant-like properties. Traditional medicinal approaches, including the use of natural products, have shown promise in addressing such conditions. In this context, *Pinus densiflora*, a species widely recognized in East Asian traditional medicine, has garnered significant attention for its potential therapeutic properties. 

*Pinus densiflora* Siebold & Zucc. is a member of the Pinaceae family, commonly referred to as the Japanese red pine. This species is native to regions such as Honshu, Shikoku, and Kyushu in Japan, with additional distribution in the Korean Peninsula and mainland China. In accordance with the pharmacopeia of the Ming Dynasty, “*Bencao Gangmu*” pine is considered a restorative agent, fortifying dental enamel, enhancing visual and auditory faculties, alleviating dermatological conditions, and conferring longevity with prolonged usage. In recent years, research has focused on antiviral (Ha et al., 2020[15]), antithrombosis (Park et al., 2016[24]), antioxidant, antimutagenic, and antitumor (Kwak et al., 2006[17]) properties. However, its potential antidepressant effects and underlying mechanisms have not been explored to date.

This study aims to investigate the antidepressant effects of hot water extracts from Pinus densiflora in a neuroinflammation mouse model. Our research focuses on elucidating the underlying mechanisms of the neuroprotective effects of pine needle (PN) through exploratory whole-transcriptomics analysis of the mouse brain. 

## Materials and Methods

### Preparation of hot water extract of pine needles 

PNs were obtained from a commercial supplier in Okayama Prefecture, Japan, in April 2023. The supplier provided species identification information. To confirm species accuracy, we also conducted morphological assessments. One gram of the dried and crushed needles was subjected to boiling with 10 mL of distilled water at 105 °C for 30 minutes in an autoclave. We selected the autoclave extraction at 105 °C specifically to maximize extraction efficiency and to obtain consistent, reproducible data. The resultant extract was subsequently filtered through a 0.2 µm membrane filter (Tosoh, Yamaguchi, Japan).

### Analysis of components in the hot water extract of pine needles

The components contained in pine needles were separated and qualified by GC/MS. To prepare a 1 g solution, 0.5 g of the extract was dissolved in methanol. The solution was centrifuged at 15,000 rpm for 5 minutes at 25 °C, and the supernatant was collected in a vial. The supernatant was used as the sample solution. The measurement conditions are shown below.

The GC/MS system we used was an Agilent 5977B GC/MSD coupled to an Agilent 7890A. The silica capillary column was a VF-5ms column (30 m × 0.25 mm i.d., film thickness 0.25 μm, Agilent Technologies Japan, Ltd., Tokyo, Japan) for separations. Helium was used as the carrier gas, and the flow rate was set to a constant flow mode of 1 mL/min. Furthermore, the oven temperature program was set to maintain 50 °C for 5 minutes, followed by a temperature ramp of 10 °C per minute up to 300 °C, and then maintained at that temperature for an additional 15 minutes. The mass spectrometer was a quadrupole type, and measurements were performed in scan mode (m/z 29-650). For each ion peak, the compound was estimated from the library and its mass spectral information. Next, a quantitative analysis of each component was performed.

Catechol and hydroquinone were quantified by creating a calibration curve based on the concentrations of standard samples and their peak areas obtained from GC/MS. The measurement parameters for GC and MS were set to the same values as those used in the qualitative analysis. For the construction of the calibration curves, catechol with a purity exceeding 99 % from FUJIFILM Wako Pure Chemical Corporation (Osaka, Japan) and hydroquinone with a purity exceeding 99 % from Tokyo Chemical Industry (Tokyo, Japan) were used, respectively.

Among the components qualitatively analyzed, D-pinitol was derivatized using a TMS reagent to convert its hydroxyl groups. A calibration curve was created based on the concentrations of standard samples and their peak areas from GC/MS for quantification. The measurement parameters for GC and MS were set to the same values as those used in the qualitative analysis. The derivatization reagent used was TMSI-C (GL Sciences Inc., Tokyo, Japan), and the derivatization treatment was performed according to the conditions specified by the reagent manufacturer. For the calibration curve, D-pinitol was provided by Tokyo Chemical Industry (Tokyo, Japan) with a purity greater than 98 % was used.

Shikimic acid quantification was performed using shikimic acid (>97 %, Tokyo Chemical Industry) as the standard for the calibration curve. The analysis was conducted on an HPLC system, specifically the Agilent 1260 Infinity II Prime LC with a PDA detector. The separation and quantification of Shikimic acid were conducted under the following HILIC conditions: An InertSustain Amide column (3.0 × 150 mm, 3 μm; GL Sciences) and a corresponding guard column were employed. The binary mobile phase consisted of solvent A (100 mM ammonium formate in water) and solvent B (acetonitrile). The gradient program was set at an isocratic condition of 20 % A and 80 % B. The flow rate was maintained at 0.5 mL/min, with the PDA detector set to monitor at 210 nm. 

Quantification of 3-hydroxybenzoic acid was performed using HPLC, like that of shikimic acid. The 3-hydroxybenzoic acid used for the construction of the calibration curve was obtained from Tokyo Chemical Industry (Tokyo, Japan) and had a purity exceeding 98 %. The column used was Poroshell 120 EC-C18 (3.0 × 150 mm, 2.7 μm; Agilent Technologies, Inc.) along with a corresponding guard column. The binary mobile phase consisted of solvent A (0.1 wt % formic acid in water) and solvent B (methanol). The flow rate was set to 0.3 mL/min, and the column temperature was maintained at 40 °C. The gradient program was configured to start with an initial concentration of 5 % for solvent B, reaching 80 % over 40 minutes using a linear gradient. Additionally, the PDA detector was set to monitor at 210 nm.

Minor components other than catechol, hydroquinone, D-pinitol, shikimic acid, and 3-hydroxybenzoic acid were quantified using catechol as the standard. The quantification was performed based on the concentrations of the standard solutions and the peak areas obtained from GC/MS, utilizing the calibration curve created accordingly.

### Cell culture

The human neuroblastoma clone SH-SY5Y cell line was obtained from the American Type Culture Collection (ATCC®, Manassas, VA, USA). The cells were cultured in a 1:1 (v/v) mixture of Dulbecco's modified Eagle's medium and Ham's F-12 medium (Gibco, Grand Island, NY, USA) supplemented with 15 % fetal bovine serum (FBS) (Sigma-Aldrich, St. Louis, MO, USA), MEM nonessential amino acids (FUJIFILM Wako Pure Chemical Corporation), and 1 % penicillin (5000 μg/mL)-streptomycin (5000 IU/mL) solution (Sigma-Aldrich) as a growth medium in 75 cm^2 ^flask. The flasks were maintained under an atmosphere of 5 % CO_2_/95 % humidified air at 37 °C. The medium was changed every two days. Serum-free Eagle's minimum essential medium (OPTI-MEM) (Gibco)was used to culture cells for the cell viability assay.

### 3-(4,5-Dimethylthiazol-2-yl)-2,5-diphenyltetrazolium bromide (MTT) assay 

The effects of PN (at dilutions of 1/20,000,1/10,000, 1/5,000, 1/2,500, 1/1,000, and 1/500) and dexamethasone (DEX) (250 μM) on cell viability were determined using the MTT assay. The MTT assay measures metabolic activity as an indirect indicator of cell viability, rather than directly measuring cell proliferation (Capes-Davis et al., 2021[7]). Furthermore, the effects of individual components present in PN, namely catechol, hydroquinone, 3-hydroxybenzoic acid, shikimic acid, and D-pinitol, were also evaluated. Specifically, since catechol is present in PN at a concentration of 0.00182M, it was diluted to achieve final concentrations of 0.091, 0.182, 0.363, 0.727, 1.817, and 3.633 μM, reflecting the dilution ratios of PN. The other components (D-pinitol, shikimic acid, hydroquinone, and 3-hydroxybenzoic acid) were prepared in an analogous manner. In addition, a mixture comprising catechol, D-pinitol, shikimic acid, hydroquinone, and 3-hydroxybenzoic acid was prepared at their respective concentrations in PN and subsequently diluted to concentrations of 1/20,000, 1/10,000, 1/5,000, 1/2,500, 1/1,000, and 1/500. SH-SY5Y cells were seeded in 96-well plates at a density of 2 × 10^5^ cells per well and incubated for 24 hours. After incubation, cells were pre-treated with PN or individual components for 1 hour, followed by treatment with 250 μM DEX for 48 hours. After the 48-hour treatment period, the medium was removed, and 100 μL of OPTI-MEM containing 0.5 mg/mL of dissolved MTT was added, followed by incubation for 24 hours (Mosmann, 1983[22]). The resulting MTT formazan was dissolved in 100 μL of 10 % sodium dodecyl sulfate (w/v), and the absorbance was measured at 570 nm using a microplate reader (Varioskan™ LUX, Thermo Fisher Scientific, Waltham, MA, USA).

### Animals and sample treatment

To evaluate the efficacy of PN, ICR mice were orally administered PN daily for 7 consecutive days. Subsequently, mice received a single intraperitoneal injection of lipopolysaccharide to induce depression-like behavior. Behavioral assessment was conducted using the tail suspension test (TST). Eight-week-old male ICR mice were purchased from Jackson Laboratory Japan (Kanagawa, Japan). The animals were housed under controlled temperature and humidity conditions with unrestricted access to food and water, maintained on a 12-hour light/dark cycle. The Ethics Animal Care and Use Committee of the University of Tsukuba approved the animal care and experimental procedures used in this study (23-328). 

After a one-week acclimatization period to the laboratory conditions, the mice were randomly assigned to five groups, with six to seven mice in each group: the control (saline administration group with saline injection), the depression model group: LPS group (saline with LPS injection), positive control (bupropion with LPS injection) and PN group (50 mg/kg body weight/day PN with LPS injection).

Bupropion (norepinephrine-dopamine reuptake inhibitor; NDRI; FUJIFILM Wako Pure Chemical Corporation. Tokyo, Japan), a well-known antidepressant drug, was used as a positive control in this study. Bupropion was dissolved in saline and administered orally at a dose of 20 mg/kg body weight daily. The control group received an equivalent volume of saline orally each day. PN was dissolved in saline and administered orally at a dose of 50 mg/kg body weight daily. The treatment duration was seven days.

### Lipopolysaccharide administration and Tail Suspension Test (TST)

Lipopolysaccharide (LPS; FUJIFILM Wako Pure Chemical Corporation), which is derived from Gram-negative bacteria, is commonly utilized to elicit a systemic immune response, inducing significant physiological and behavioral alterations referred to as 'sickness behavior.' This syndrome encompasses symptoms such as anhedonia, lethargy, decreased appetite, heightened anxiety, and increased drowsiness. LPS was dissolved in saline and administered as a single intraperitoneal injection at a dose of 0.85 mg/kg body weight. 

On the seventh day of oral administration, one hour after the final oral administration, LPS was administered intraperitoneally. Twenty-four hours following the intraperitoneal injection of LPS into the ICR mice, the tail suspension test (TST) was conducted as described in a previous study (Sasaki et al., 2021[27]) with slight modifications. Briefly, each mouse was individually suspended for 6 minutes at a point 2 cm from the tail tip using a clip (Yamashita Giken, Tokushima, Japan) in a white box measuring 30 × 15 × 50 cm (length × width × height). The immobility time, indicative of behavioral despair akin to clinical depression in humans, was recorded and measured during the final 4 minutes of the 6-minute suspension period using SMART 3.0 video tracking software (Panlab, Barcelona, Spain). Mice were considered immobile only when they ceased movement of their limbs, head, and body.

### Hippocampus isolation from mice brains

After the final TST on the seventh day, all the mice were euthanized by cervical dislocation. The whole brain of each mouse was quickly removed, and the hippocampus was carefully dissected on ice and rapidly frozen in liquid nitrogen. Total RNA was extracted from the hippocampus using an ISOGEN (Nippon Gene Co. Ltd., Tokyo, Japan) following the manufacturer's instructions. Total RNA concentration was determined using a NanoDrop 2000 spectrophotometer (Thermo Fisher Scientific, Wilmington, DE, USA).

For protein extraction, the hippocampus (50-100 mg) was homogenized in 1 mL of radioimmunoprecipitation assay (RIPA) (Sigma-Aldrich) buffer with a protease inhibitor (Sigma-Aldrich). The homogenate was centrifuged at 10,000 × g for 30 minutes at 4 °C, and the supernatant was collected for protein analysis.

### Microarray analysis

Microarray analysis was performed on RNA samples extracted from the hippocampus of mice subjected to the TST, control (water treatment), and 50 mg/kg PN administration groups. The Clariom S assay Mouse system (Thermo Fisher Scientific K.K., Tokyo, Japan) was used for triplicate RNA samples from each group. According to the user manual, cDNA for microarray analysis was generated using a GeneChip WT Plus Reagent kit (Thermo Fisher Scientific K.K.). The samples were hybridized using a Clariom S GeneChip Microarray Kit for mice (Thermo Fisher Scientific K.K.). After washing and staining, images were captured, and raw intensity data were generated using a GeneChip Scanner 3000 (Thermo Fisher Scientific K.K.).

### Microarray data processing and data analyses

Raw image data processing and normalization were performed using Transcriptome Analysis Console (TAC) software version 4.0.2 (Thermo Fisher Scientific, MA, USA) following the signal space transformation robust multi-chip analysis (SST-RMA) algorithm. The gene-level analysis was then performed employing the Limma Bioconductor package included with TAC 4.0.2. A one-way ANOVA was applied to identify the differentially expressed genes (DEGs), followed by an empirical Bayes correction to enhance the statistical reliability of the results.

A detected above background (DABG) cutoff of 0.05 was set to refine the analysis further to eliminate non-significant signals. Additionally, the positive vs. negative area under the curve (AUC) value was conservatively set at greater than or equal to 0.7 to focus on genes with strong discriminatory potential.

The final selection of DEGs was based on stringent filter criteria, requiring a p-value of less than 0.05 (calculated using one-way between-subject ANOVA) and −1.2 > linear fold change > 1.2.

Volcano plots were generated using the VolcaNoseR web application (Goedhart and Luijsterburg, 2020[14]).

Gene ontology (GO) enrichment analyses were conducted using the web-based tool Metascape v3.5.20240101 (http://metascape.org) (accessed on 19 Jan 2024) (Zhou et al., 2019[44]).

The SynGO web tool was utilized to explore overrepresented synaptic GO terms and to generate sunburst plots (https://www.syngoportal.org/). SynGO is an open-access knowledge base dedicated to synapse research, providing approximately 3,000 annotations related to synapse-specific protein location or function and about 1,100 distinct genes/proteins (Koopmans et al., 2019[16]).

Network Analyst version 3.0 was employed to construct protein-protein interaction (PPI) networks (Zhou et al., 2019[43]). The first-order PPI network was built based on the IMEx Interactome database, which consists of comprehensive, literature-curated data sourced from InnateDB (Breuer et al., 2013[5]). Kinase enrichment analysis was conducted on the KEA3 web tool. 

### Quantitative Real-Time PCR

Real-time PCR was conducted to analyze gene expressions in the hippocampus of mice. The TaqMan probe (Thermo Fisher Scientific) was used for gene expression quantification. A cDNA solution was synthesized using a superscript IV VILO master mix (Thermo Fisher Scientific) following the manufacturer's instructions. For the quantification of transcript levels, TaqMan real-time RT-PCR amplification reactions were performed using the Applied Biosystems 7500 Fast Real-Time PCR System (Thermo Fisher Scientific). All primer sets and the TaqMan Universal PCR Master Mix were obtained from Thermo Fisher Scientific. *Gapdh* (Mm99999915_g1) was used as an internal control, and the following targets: *Bdnf* (Mm04230607_s1), *Tnf* (Mm00443258_m1), *Il1b* (Mm00434228_m1), *Apln* (Mm00443562_m1), and *Aplnr* (Mm00442191_s1).

### ELISA analysis

The concentrations of neurotransmitters were evaluated employing an ELISA kit (ImmSmol, Talence, France). Specifically, the neurotransmitters dopamine (DA) (immuSmol BA-E-5300R), norepinephrine (NA) (immuSmol BA-E-5200R), and serotonin (5-HT) (immuSmol BA E-5900R) were quantified. Furthermore, the levels of mature brain-derived neurotrophic factor (mature BDNF) were determined using an ELISA kit (Biosensis, BEK-2211-1, Thebarton, South Australia), in accordance with the manufacturer's guidelines. In summary, the concentrations of 5-HT, NA, DA, and mature BDNF in the brain were measured utilizing protein samples or standards. Following treatment with antibodies, a secondary incubation with streptavidin-horseradish peroxidase conjugate solution was conducted for 60 minutes. After the addition of substrate and stopping solution, the levels of 5-HT, NA, DA, and mature BDNF were assessed by measuring absorbance at 450 nm; these levels were subsequently normalized to protein concentration using the Pierce BCA Protein Assay (Thermo Fisher Scientific).

### Statistical analysis

Data were analyzed using GraphPad Prism (version 10, GraphPad Software Inc., San Diego, CA). Data are expressed as mean ± standard error of the mean (SEM) unless otherwise noted. Shapiro-Wilk test was conducted to determine the normal distribution of the continuous variables. For comparisons among multiple groups, one-way analysis of variance (ANOVA) was employed, followed by Dunnett's post hoc test. Differences were considered statistically significant at a value of P < 0.05.

## Results

### PN ameliorated LPS-induced depressive-like behavior in ICR mice

**Figure 2:**
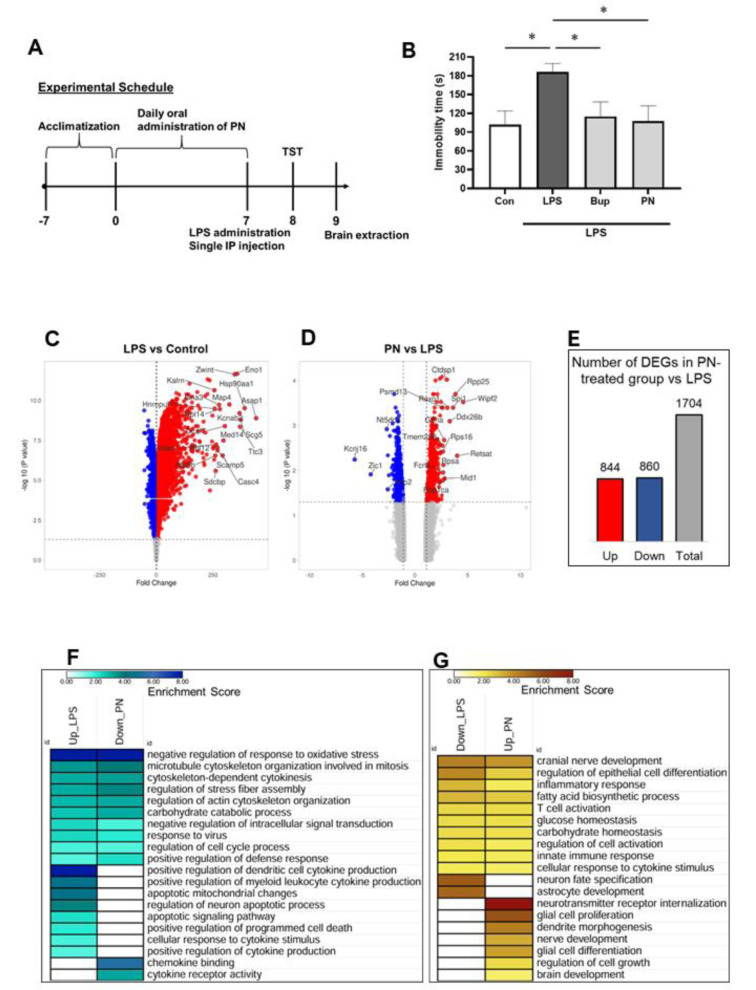
Evaluation of the effects of PN on depressive behavior, using the tail suspension test, in mice induced with LPS. A) Schedule for the Behavioral Test to Evaluate the Effects of PN on LPS-Induced Neuroinflammatory Mice. B) The immobility time in tail suspension test. The data are expressed as mean ± SEM. *p < 0.05 vs LPS group, by one-way ANOVA followed by Dunnett's post hoc test. n =6~7. Volcano plots displaying differentially expressed genes (DEGs) between C) LPs-induced vs Control mice and D) PN-treated vs LPs-induced mice. The vertical axis (y-axis) corresponds to −log10 p-value and the horizontal axis (x-axis) displays linear fold change. The red dots represent the upregulated genes; the blue dots represent the downregulated genes. The top 20 DEGs with the biggest fold changes are shown. E) Column graph showing the number of DEGs in PN-treated mice compared to the LPS-induced group. The red bar represents the number of upregulated DEGs, the blue bar represents the number of downregulated DEGs, and the grey bar represents the total number of DEGs. The heatmap shows significantly enriched gene ontology terms: F) Terms that were upregulated in the LPS-induced group but downregulated by PN treatment. G) Terms that were downregulated in the LPS-induced group but upregulated by PN treatment. The color bar represents the enrichment score, with darker colors indicating higher enrichment.

As shown in Figure 2A(Fig. 2), treatment with PN significantly reduced the immobility time in the tail suspension test compared to the LPS-only group, indicating an antidepressant-like effect. Bupropion was used as a positive control and showed similar effects.

LPS-injected ICR mice demonstrated a significant increase in immobility time (186.13 ± 13.26 s, p = 0.016) in comparison to the saline-administered control group (101.95 ± 21.63 s). Conversely, oral administration of PN at a dosage of 50 mg/kg for seven days prior to the LPS injection significantly attenuated the LPS-induced increase in immobility time (107.49 ± 24.29 s, p = 0.026), which was even lower than that observed in the positive control Bupropion group (114.95 ± 23.26 s, p = 0.049 vs LPS group) (Figure 2B(Fig. 2)). Throughout the study, we monitored the body weight, overall health status of the animals, and the quantity of food consumed, revealing no significant differences among the groups (data not shown).

### PN treatment regulated gene expression in TST stress-induced mice hippocampus

Next, we performed whole-genome microarray analysis to determine the molecular mechanism underlying the anti-stress effects of PN observed in the behavioral test. A total of 22,206 probe IDs were identified in the Clariom S Assay Mouse Array. The comparison between the LPS group and the control group revealed 17,829 differentially expressed genes (DEGs), including 9,841 upregulated and 7,988 downregulated genes. The top 20 differentially expressed genes (DEGs) with the largest fold changes (FC) are pointed out on the volcano plots. In the volcano plots, upregulated genes are represented by red dots, whereas blue dots denote downregulated genes. Notably, the top upregulated DEG was ArfGAP with SH3 domain, ankyrin repeat and PH domain1 (*Asap1*, FC = 440.93), while the top downregulated DEG was leucine rich repeat containing 66 (*Lrrc66*) with FC = −53.74 (Figure 2C(Fig. 2)). In comparison to the LPS group, PN exhibited 1704 differentially expressed genes (DEGs), with 844 being upregulated and 860 being downregulated (Figure 2D, 2E(Fig. 2)). Notably, the top upregulated DEG was WAS/WASL interacting protein family, member 2 (*Wipf2*, FC = 4.57), while the top downregulated DEG was potassium inwardly-rectifying channel, subfamily J, member 16 (*Kcnj16*, FC =−5.72) in PN group (Figure 2D(Fig. 2)).

### PN treatment could reverse LPS-induced biological events in mice hippocampus

We compared the terms that were upregulated under LPS vs control conditions with those that were downregulated under PN vs LPS conditions. The common term that was enriched in both comparisons was negative regulation of response to oxidative stress (GO:1902883), microtubule cytoskeleton organization involved in mitosis (GO:1902850),cytoskeleton-dependent cytokinesis (GO:0061640), regulation of stress fiber assembly (GO:0051492), regulation of actin cytoskeleton organization (GO:0032956), carbohydrate catabolic process (GO:0016052), negative regulation of intracellular signal transduction (GO:1902532), response to virus (GO:0009615), regulation of cell cycle process (GO:0010564) and positive regulation of defense response (GO:0031349). Specifically, the term that was upregulated in the LPS vs control conditions was positive regulation of dendritic cell cytokine production (GO:0002732), positive regulation of myeloid leukocyte cytokine production involved in immune response (GO:0061081), apoptotic mitochondrial changes (GO:0008637), regulation of neuron apoptotic process (GO:0043523), apoptotic signaling pathway (GO:0097190), positive regulation of programmed cell death (GO:0043068), cellular response to cytokine stimulus (GO:0071345) and positive regulation of cytokine production involved in immune response (GO:0002720), whereas the term that was downregulated in the PN versus LPS conditions was chemokine binding (GO:0019956) and cytokine receptor activity (GO:0004896) (Figure 2F(Fig. 2)).

Next, we compared the terms that were downregulated under LPS vs control conditions with those that were upregulated under PN vs LPS conditions. The common term that was enriched in this analysis was cranial nerve development (GO:0021545), regulation of epithelial cell differentiation (GO:0030856), inflammatory response (GO:0006954), long-chain fatty acid biosynthetic process (GO:0042759), T cell activation (GO:0042110), glucose homeostasis (GO:0042593), carbohydrate homeostasis (GO:0033500), regulation of cell activation (GO:0050865), innate immune response (GO:0045087) and cellular response to cytokine stimulus (GO:0071345). In this case, the term that was downregulated in the LPS vs control conditions was neuron fate specification (GO:0048665) and astrocyte development (GO:0014002), while the term that was upregulated in the PN vs LPS conditions was neurotransmitter receptor internalization (GO:0099590), glial cell differentiation (GO:0010001), dendrite morphogenesis (GO:0048813), nerve development (GO:0021675), glial cell proliferation (GO:0014009), regulation of cell growth (GO:0001558) and brain development (GO:0007420) (Figure 2G(Fig. 2)).

### PN regulated apelin signaling pathway in mice hippocampus

**Figure 3:**
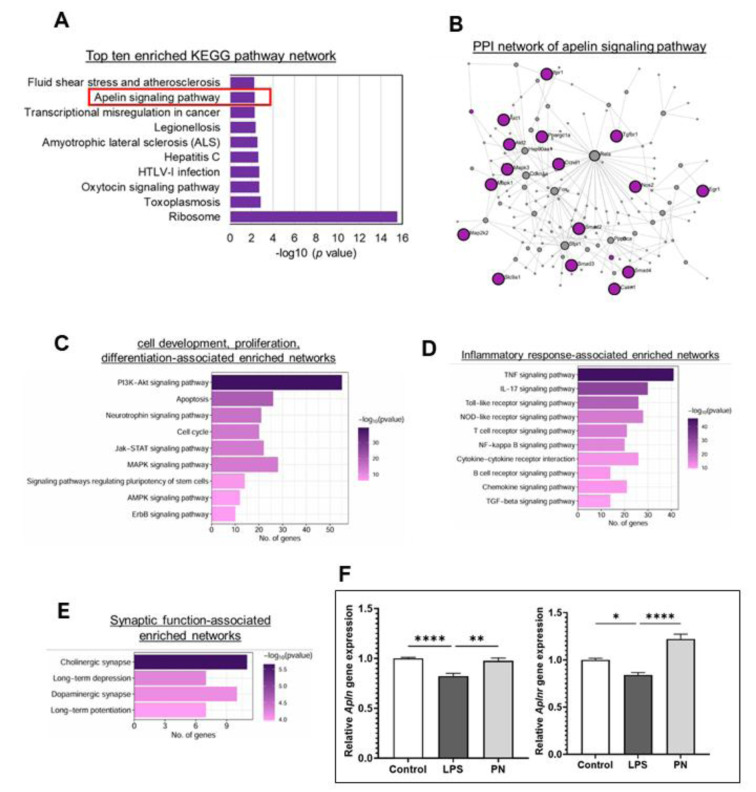
Effects of PN on apelin signaling pathway. A) Bar graph displaying the top ten significantly enriched KEGG pathway networks based on upregulated DEGs in the PN-treated group compared to the LPS group. The x-axis represents significance (-log10 p-value). B) PPI analysis illustrating gene interactions related to the apelin signaling pathway among DEGs in PN-treated conditions. Purple-colored nodes indicate significant DEGs directly associated with the apelin signaling pathway, while grey-colored nodes represent closely linked genes within the pathway. The top enriched functional networks within the apelin signaling pathway are shown in the following bar plots: C) Cell development, proliferation, and differentiation-associated network; D) Inflammatory response-associated network; E) Synaptic function-associated network. The color code represents significance (-log10 p-value) of enriched terms, while the x-axis refers to number of interacting genes within each term. F) mRNA expression levels of Apln and its receptor Aplnr as determined by real-time qPCR (n = 4~). *p < 0.05, **p < 0.01, ****p < 0.001, by one-way ANOVA followed by Dunnett's post hoc test.

We conducted a PPI analysis to explore the interactions among the DEGs in the treatment groups, aiming to identify potential signaling pathways. The bar graph displays the top ten significantly enriched KEGG pathway networks based on upregulated DEGs in the PN-treated group compared to the LPS group. Notably, the Ribosome pathway was enriched, along with the Apelin signaling pathway (Figure 3A(Fig. 3)).

PPI analysis shows Apelin signaling pathway-related gene interaction among the DEGs in PN-treated conditions (Figure 3B(Fig. 3)). Purple color nodes are the significant DEGs directly associated with the apelin signaling pathway, while the grey-colored nodes are closely linked genes with the apelin signaling pathway. 

We present the top enriched functional networks within the apelin signaling pathway in the bar plots. Notably, networks associated with cell development, proliferation, and differentiation; inflammatory responses; and synaptic function were enriched (Figure 3C-E(Fig. 3)).

Subsequently, we assessed the effects of PN administration on apelin and apelin receptor expression in the hippocampus of LPS-induced mice utilizing RT-qPCR analysis (Figure 3F(Fig. 3)). The results indicated that LPS administration significantly decreased the expression of both apelin (*Apln*; 0.82 ± 0.028; p < 0.0001) and its receptor (*Aplnr*, 0.84 ± 0.024; p = 0.018) compared to the control group (1.00 ± 0.0088, 1.00 ± 0.016, respectively). Nevertheless, in the PN group, there was a significant reversal of this decline (0.97 ± 0.17; p = 0.024, 1.22 ± 0.050; p < 0.0001, respectively).

### PN increased neurotransmitters in TST stress-induced mice model

**Figure 4:**
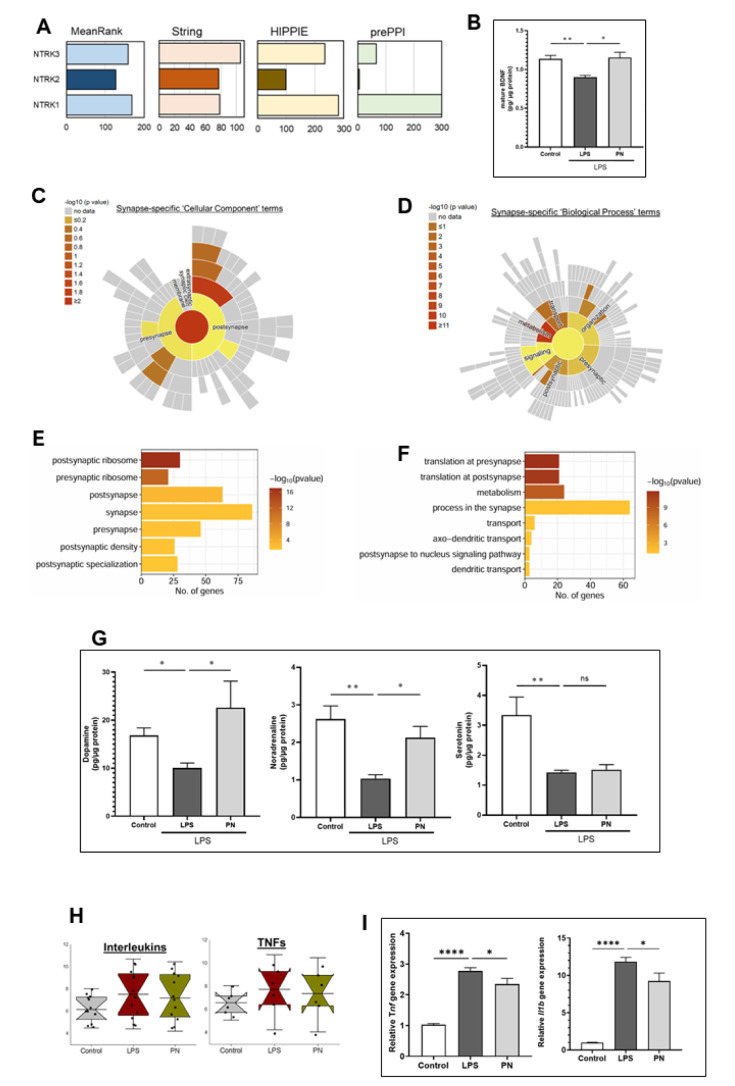
Key biological events regulated by PN in mice hippocampus. A) Kinase enrichment analysis was performed to identify upstream NTRKs whose putative substrates are overrepresented in the upregulated DEGs of the PN-treated group compared to the LPS-induced group. The column graphs display the rankings of all three NTRKs-NTRK1 (TRKA), NTRK2 (TRKB), and NTRK3 across three different protein-protein interaction (PPI) network databases: STRING, HIPPIE, and prePPI. Additionally, an integrated ranking (MeanRank) is provided. A lower rank indicates a higher level of interaction. Among these, NTRK2 (TRKB), the receptor for brain-derived neurotrophic factor (BDNF), exhibited the highest level of interaction in the PN-treated group. B) mature BDNF protein expressions by ELISA were examined (n = 4~). *p < 0.05, **p < 0.01, by one-way ANOVA followed by Tukey's post hoc test. Sunburst plots illustrate the overrepresented synaptic terms among the upregulated DEGs in the PN-treated group: C) Cellular component (location); D) Biological process (function). The color bar represents significance (-log10 p-value). Bar graphs display the overrepresented terms in: E) Cellular component; F) Biological process. The color bar indicates significance (-log10 p-value), while the x-axis represents the number of upregulated DEGs. G) Expression levels of the neurotransmitters dopamine, noradrenaline, and adrenaline were measured using ELISA (n = 4~). p < 0.05, p < 0.01, analyzed by one-way ANOVA followed by Dunnett's post hoc test. H) Average signal intensities of interleukins and TNF mRNAs in the microarray dataset. I) mRNA expression levels of *Tnf *and *Il1b* as determined by real-time qPCR (n = 4~). *p < 0.05, ****p < 0.001, by one-way ANOVA followed by Dunnett's post hoc test.

We investigated the key biological events occurring in the hippocampus of mice following PN treatment. First, a kinase enrichment analysis was performed to identify upstream NTRKs whose putative substrates are overrepresented in the upregulated differentially expressed genes (DEGs) of the PN-treated group compared to the LPS-induced group. The bar graphs display the rankings of all three NTRKs-NTRK1 (TRKA), NTRK2 (TRKB), and NTRK3-across three different protein-protein interaction (PPI) network databases: STRING, HIPPIE, and prePPI. Additionally, an integrated ranking (MeanRank) is provided, where a lower rank indicates a higher level of interaction. Among these, NTRK2 (TRKB), the receptor for brain-derived neurotrophic factor (BDNF), exhibited the highest level of interaction in the PN-treated group (Figure 4A(Fig. 4)). Subsequently, we quantified levels of mature brain-derived neurotrophic factor (BDNF) in the hippocampus of mouse brains to further investigate the underlying mechanisms that may contribute to the antidepressant-like effects of PN. Utilizing commercially available ELISA kits, our findings indicated that mature BDNF levels (1.15 ± 0.07 pg/μg protein, p = 0.011) were significantly elevated in the PN group when compared to the LPS group (0.90 ± 0.03, pg/μg protein) (Figure 4B(Fig. 4)).

We subsequently performed synapse-specific Gene Ontology biological process (GOBP) analysis utilizing the web-based tool Synaptic Gene Ontologies (SynGo), which is adept at analyzing synapse-specific cellular components (CCs) (Figure 4C(Fig. 4)) and biological processes (BPs) (Figure 4D(Fig. 4)). The analysis encompassed 727 upregulated genes in the PN group, of which 90 were annotated by SynGO. In the cellular component category, significant enrichment was observed in “presynaptic ribosomes” and “postsynaptic ribosomes.” In the biological process category, “metabolism” was markedly enriched. Bar graphs display the overrepresented terms in cellular component and biological processes (Figure 4E and F(Fig. 4)). For the downregulated genes in PN, no significantly enriched gene sets were identified.

We quantified levels of dopamine (DA), noradrenaline (NA), and serotonin (5-HT) in the hippocampus of mouse brains to further investigate the underlying mechanisms that may contribute to the antidepressant-like effects of PN. Utilizing commercially available ELISA kits, our findings indicated that DA (22.60 ± 5.54 pg/μg protein, p = 0.015) and NA (2.13 ± 0.30 pg/μg protein, p = 0.036), were significantly elevated in the PN group when compared to the LPS group (10.08± 0.99, 1.04 ± 0.10 pg/μg protein, respectively). In contrast, 5-HT levels (1.52 ± 0.17 pg/μg protein, p > 1.0) did not demonstrate any significant difference compared to the LPS group (1.43± 0.07 pg/μg protein) (Figure 4G(Fig. 4)).

### PN alleviated the mRNA expression of pro-inflammatory cytokines expression in the hippocampus of mice induced by LPS

Further analysis of the signal intensity of interleukins and TNF probe IDs across all three groups (control, LPS, and PN+LPS) revealed an increase in signal intensity in the LPS condition relative to the control condition. Nevertheless, the signal intensity of interleukins and TNFs was reduced in the PN-treated condition compared to the LPS group (Figure 4H(Fig. 4)).

Next, we validated the effects of PN administration on pro-inflammatory cytokines in the hippocampus of LPS-induced mice using RT-qPCR analysis (Figure 4I(Fig. 4)). The administration of LPS resulted in a significant elevation in *Tnfa* mRNA expression (2.78 ± 0.099; p < 0.0001) compared to the control group (1.03 ± 0.033). However, this increase was remarkably attenuated in the PN group (2.35 ± 0.18; p = 0.024). Likewise, LPS administration significantly augmented *Il1b* expression (11.85 ± 0.57; p < 0.0001) in comparison to the control group (1.00 ± 0.035), which was significantly mitigated in the PN group (9.24 ± 1.05; p = 0.014). These results imply that PN may alleviate LPS-induced neuroinflammation in the mouse brain.

### Components analysis

**Figure 5:**
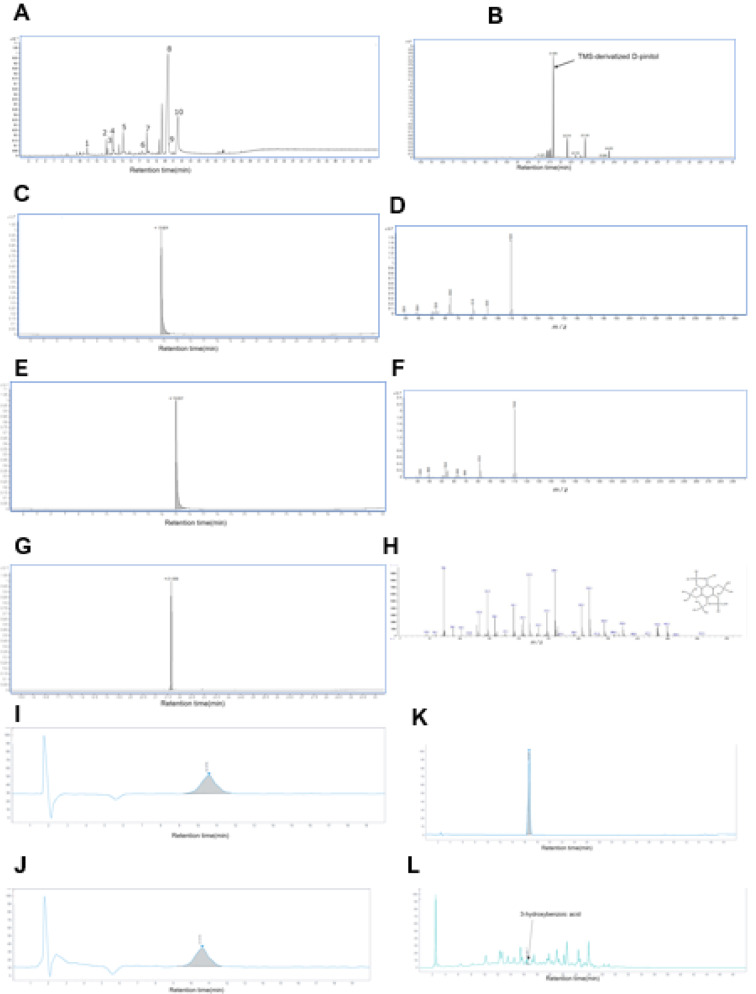
Analysis of PN components. A) The chromatogram obtained from gas chromatography-mass spectrometry (GC/MS) analysis, depicting the components contained in PN. B) GC/MS chromatogram of TMS-derivatized D-Pinitol, illustrating its retention time and detection within the PN sample analysis. C) GC/MS chromatogram of catechol, highlighting its peak for subsequent spectral analysis. D) Mass spectrum of catechol, confirming its molecular structure based on characteristic fragmentation patterns. E) GC/MS chromatogram of hydroquinone. F) Mass spectrum of hydroquinone. G) GC/MS chromatogram of TMS-derivatized D-Pinitol at a concentration of 200 μg/mL. H) Mass spectrum of TMS-derivatized D-Pinitol. I) HPLC chromatogram of shikimic acid at a concentration of 2000 μg/mL. J) HPLC chromatogram detecting shikimic acid in the hot water extract of PN, obtained under condition optimized for shikimic acid analysis. K) HPLC chromatogram of 3-hydroxybenzoic acid at 50 μg/mL, demonstrating its retention time and detection profile. L) HPLC chromatogram of 3-hydroxybenzoic acid in the hot water extract of PN, acquired under condition optimized for 3-hydroxybenzoic acid detection.

GC/MS analysis of PN was conducted to qualitatively and quantitatively assess ten peaks (Figure 5A(Fig. 5)). The quantitative measurements were standardized using catechol. The identified compounds included Glycerin (retention time = 9.63min, 40 ppm), 2,3-Dihydro-3,5-dihydroxy-6-methyl-4H-pyran-4-one (retention time = 13.11 min, 69 ppm), Benzoic acid (retention time = 13.28 min, 58 ppm), Catechol (retention time = 13.88 min, 170 ppm), Hydroquinone(retention time = 15.09 min, 209 ppm), Sucrose (retention time = 17.33 min, 58 ppm), Hydroxybenzoic acid (retention time = 17.87 min, 188 ppm), D-Pinitol (retention time = 20.37 min, 3208 ppm), Inositol (retention time = 20.43 min, 71 ppm) and shikimic acid (retention time = 21.53 min, 1236 ppm) (Table 1(Tab. 1)). Additionally, the major components, D-Pinitol, shikimic acid, catechol, hydroquinone, and hydroxybenzoic acid were quantified using their respective standards, yielding concentrations of 2376 ppm (0.0122 M), 5930 ppm (0.0341 M), 200 ppm (0.00182 M), 162 ppm (0.00147M) and 5 ppm (36.2μM), respectively (Figure 5B-L(Fig. 5)).

### Effects of components in PN on cell viability and neuroprotection in human neuroblastoma SH-SY5Y cells

**Figure 6:**
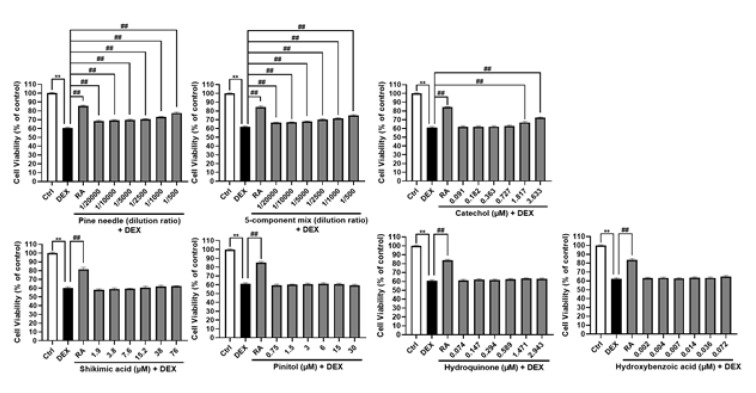
Evaluation of neuroprotective effects against dexamethasone (DEX) in SH-SY5Y cells for each component of PN and their respective mixtures. Statistical significance was assessed using one-way ANOVA followed by Dunnett's post-hoc test. Each bar represents the mean ± SE (n=5 (Pine needle), n=3 (Mix, Catechol, Hydroquinone, and Hydroxybenzoic acid), and n =2 (Shikimic acid, and Pinitol). **p < 0.01 vs. control cells, ##p < 0.01 vs. DEX-treated cells.

We confirmed the neuroprotective effect of PN components against dexamethasone (DEX) in SH-SY5Y cells. A previous study showed that 500μM DEX induces cytotoxicity in SH-SY5Y cells (Omari et al., 2021[23]). The treatment concentration of PN was determined via MTT assay (Supplementary information). When mixed with five other components, similar neuroprotective effects to PN were observed, particularly with catechol at a dilution of 1/1000 (1.817 μM). In contrast, no neuroprotective effects were noted for D-pinitol, shikimic acid, hydroquinone, or 3-hydroxybenzoic acid (Figure 6(Fig. 6)). 

## Discussion

In this study, we conducted a series of investigations in both in vitro and in vivo settings to elucidate the mechanisms underlying the antidepressant effects of PN. This is the first report on PN regulating the apelin signaling pathway in a depression-like mouse model, which in turn alleviates neuroinflammation, enhances BDNF production, and improves the production of neurotransmitters.

First, we examined the antidepressant-like effects of PN on the behavioral profiles of TST-stimulated depression mice. The TST is one of the most widely used models to assess antidepressant-like activity in rodents and has been suggested to be more sensitive than other behavioral tests, such as the forced swimming test. The TST behavioral paradigm is standardized and comprehensive for studying the neurobiological mechanisms of depression. Our results indicated that oral administration of 50 mg/kg PN for seven days significantly reduced immobility time in mice when compared to stress-induced saline-treated mice, suggesting the antidepressant-like properties of PN.

In summary, our findings provide evidence that oral administration of PN induces an increase in neuro-metabolism and enhances the expression of cell proliferation, differentiation, and neurotransmitter-related genes while decreasing the expression of neuroinflammation-related genes.

Specifically, our DNA microarray analysis suggested that PN may activate the apelin signaling pathway. Additionally, our study demonstrated that PN-treated mice exhibited increased expression of Apelin and its receptor in the hippocampus. The endogenous ligand for the APJ receptor (APLNR), Apelin (APLN), was initially isolated from bovine gastric tissue. The Apelin peptide is synthesized in vivo as a precursor comprising seventy-seven amino acid residues, cleaved by endopeptidases to yield the active Apelin-13 peptide consisting of thirteen amino acids (Tatemoto et al., 1998[30]). The role of the Apelin/APJ system in the nervous system is particularly intriguing. Apelin-immunoreactive neuronal cell bodies are distributed throughout the arcuate nucleus of the hypothalamus, indicating a potential role in neuroendocrine functions (Goazigo et al., 2011[13]). Depression has a complex origin, and the Apelin/APJ system is currently understood to engage in crosstalk through neuroendocrinology, neurotrophic factors, and inflammation. Notably, Apelin-13, a key neuropeptide known for its inhibitory effects on neuroinflammatory processes and oxidative stress in the brain, demonstrates beneficial properties in mitigating memory impairment and neuronal injury (Mohseni et al., 2021[21]; Yildiz et al., 2021[38]). 

Recent studies have indicated that pine needles possess antioxidant and anti-apoptotic properties in rat models fed a high-cholesterol diet (Seo et al., 2014[29]), and polyphenols derived from them have been suggested to improve cognitive impairments in D-galactose-induced mouse models (Wang et al., 2014[35]). Extracts from *P. densiflora* leaves have been shown to possess a strong anti-amnesic effect against memory impairment caused by cholinergic dysfunction (Lee et al., 2017[18]). Therefore, in our study, we investigated the antidepressant effects of *P. densiflora* leaf extracts, including their mechanisms. The component analysis identified our PN extract sample is rich in shikimic acid D-pinitol, catechol, hydroquinone, or 3-hydroxybenzoic acid. Shikimic acid is known for its anti-neuroinflammatory and neuroprotective properties (Bao et al., 2023[3]; Rabelo et al., 2015[25]). Additionally, D-pinitol has been identified in soybeans, chickpeas, and lentils, and its neuroprotective effects have been confirmed (Alonso-Castro et al., 2019[1]). Shikimic acid was found to be abundant in PN. It has been reported that shikimic acid is metabolized by gut microbiota to generate catechol (Brewster et al., 1978). Therefore, it is suggested that the shikimic acid contained in PN may likewise be metabolized into catechol. Additionally, we confirmed the neuroprotective effect of catechol, a component of PN, which is recognized for its properties against oxidative stress (Troadec et al.,, 2001[33]) and inflammation (Funakoshi-Tago et al., 2020[12]; Zheng et al., 2008[42]) induced by lipopolysaccharide (LPS). Hydroquinone also exhibits neuroprotective effects through its antioxidant and anti-inflammatory properties (Troadec et al., 2001[33]). Additionally, hydroxybenzoic acid is known for its antioxidant effects (Bendini et al., 2007[4]).

Recent research has also emphasized the involvement of neurotrophic factors in the onset of depression. BDNF, as a member of the neurotrophic factor family, is an important indicator of antidepressant treatment in clinical and animal models (Saral et al., 2021[26]). Apelin-13 activates the PI3K/Akt signaling pathway associated with BDNF/TrkB activation (Li et al., 2016[19]). Specifically, the therapeutic effects of Apelin-13 on depression and memory deficits in rats in the forced swimming test (FST) are blocked by LY294002 (a PI3K inhibitor) or PD98059 (an ERK1/2 inhibitor). In short, the Apelin/APJ system plays a crucial role in the neuroprotection mediated by BDNF/PI3K/Akt signaling pathways (Faridvand et al., 2019[11]). 

Conversely, inflammatory processes also play a significant role in depression. There is abundant evidence showing the role of the Apelin/APJ system in alleviating stress and inflammation (Zhang et al., 2019[41]). The etiology and treatment of depression involve complex brain mechanisms that are strongly correlated with both neuroendocrine disorders and neuroinflammatory states. Apelin alleviates the onset of depression through its function in promoting BDNF production, anti-inflammatory, and anti-oxidative mechanisms. 

Additionally, PN treatment significantly reduced the expression of major inflammatory cytokines, TNF and IL-1β, in the hippocampus of mice. Importantly, subsequent protein expression analyses demonstrated the upregulation of mature BDNF in the hippocampus. BDNF is a member of the neurotrophin family essential for various brain functions including neurogenesis, neuron survival, learning, memory, and synaptic plasticity (Arancio and Chao, 2007[2]). BDNF is implicated in the pathophysiology of neuropsychiatric disorders, including depression (Castrén and Kojima, 2017[8]), anxiety disorders (Wang et al., 2015[36]), and schizophrenia (Zhang et al., 2012[40]). Notably, BDNF is the most representative neurotrophin associated with depression. Therefore, we quantified BDNF levels in this study. In our previous study, we obtained results indicating the involvement of neuroinflammation in modulating the BDNF/TrkB pathway, contributing to antidepressant effects (Wakasugi et al., 2024[34]). We discovered that oral administration of PN elevated BDNF levels in the hippocampus of TST-stressed mice. The activation of inflammatory cytokines is linked to decreased mRNA expression of neurotrophins like BDNF in brain structures associated with plasticity (Zhang et al., 2012[40]). In vivo studies have shown that the administration of inflammatory cytokines or LPS significantly reduces mature BDNF levels in brain regions such as the hippocampus and cerebral cortex (Zhang et al., 2016[39]). Furthermore, numerous studies suggest a potential role for BDNF in depression, influencing neurogenesis and synaptic plasticity (Yang et al., 2020[37]).

Additionally, it was observed that levels of DA, NA, and 5-HT were upregulated in the hippocampus of PN-treated mice. Within the brain, DA and NA are synthesized by specific DA neurons and play several pivotal roles through four distinct pathways: the mesolimbic, mesocortical, nigrostriatal, and tuberoinfundibular pathways. These pathways are associated with mood regulation, cognitive function, and motor function. Previous studies have reported that dysregulation of this dopaminergic system may lead to depression (Dailly et al., 2004[10]), memory deficits (Sawaguchi et al., 1988[28]), and impaired motor control. 5-HT participates in various physiological, emotional, and cognitive functions (Lucki, 1998[20]), and dysfunction of the 5-HT system is implicated in several psychiatric and neurological disorders, including depression (Cowen and Browning, 2015[9]). Thus, our findings from the quantification of neurotransmitters and microarray analysis indicate that oral administration of PN may improve the production, secretion, and transmission of neurotransmitters in TST-stressed mice. Moreover, Apelin-13 demonstrated anxiolytic effects in the elevated plus maze, and its anti-anxiety properties were mediated through α-adrenergic, β-adrenergic, dopaminergic, and 5-HT2 serotonergic receptors (Telegdy et al., 2013[31]). Additionally, apelin-13 facilitated memory consolidation in a passive avoidance paradigm in mice, with several neurotransmitters, including α-adrenaline, serotonin, acetylcholine, dopamine, and GABA, playing crucial roles in the process (Telegdy et al., 2014[31]).

Our research emphasizes the potential antidepressant-like function of PN in promoting neurogenesis and alleviating neuroinflammation. However, depression is a multifaceted condition with various accompanying pathologies. To fully confirm the effects of PN on depression and anxiety disorders, further research is required to analyze the mechanisms by which PN induces the expression of apelin and to identify specific components within PN that may interact with apelin receptors.

Our research is the first to explore the ability of PN to activate Apelin and its receptor, demonstrating its capacity to promote neurogenesis and mitigate neuroinflammation in both in vitro and in vivo models. Apelin has also been noted for its antioxidant properties. Furthermore, catechol, a component of PN, possesses antioxidant effects that may contribute to these neuroprotective and antidepressant outcomes. Thus, it is possible that catechol exerts its neuroprotective and antidepressant effects through the modulation of Apelin signaling. In summary, our findings suggest that PN represents a promising innovative therapeutic approach targeting depression-like psychiatric disorders.

While our study provides initial evidence supporting the potential neuroprotective and antidepressant-like effects of Pinus densiflora needle (PN) extract, several limitations should be acknowledged. First, further research is needed to comprehensively elucidate the efficacy and underlying mechanisms of PN extract. Future studies should incorporate long-term treatment protocols, dose-response analyses, and the inclusion of both male and female subjects to assess potential sex-specific differences in gene expression and behavioral outcomes.

Although our pathway enrichment analyses highlighted the apelin signaling pathway as a potential mediator of PN's effects, direct experimental validation through mechanistic studies is necessary to confirm this involvement. Moreover, to date, no epidemiological studies have demonstrated an association between PN consumption and reduced risk of neuropsychiatric disorders. Establishing such evidence through population-based research would be valuable in assessing the therapeutic relevance of PN and its potential health benefits in humans.

## Conclusions

In conclusion, the findings of this study clearly indicate that hot water extracts from pine needles (PN) activate the Apelin signaling pathway, alleviating neuroinflammation and promoting neurogenesis in models of depression. By enhancing the production and secretion of key neurotransmitters while reducing inflammatory markers, PN presents a novel potential intervention for treating depression-like symptoms. Further exploration is necessary to completely understand the multifaceted roles of PN in addressing depression and related neuropsychiatric disorders.

## Declaration

### Funding

This work was partially supported by JST Grant Number JPMJPF2017.

### Conflict of interest

HIO and EY are employed at (Arakawa Chemical Industries, Ltd.). Other authors declare no conflict of interest.

### CRediT author statement

**Hisako Iwahashi Ogawa**: conceptualization, methodology, validation, formal analysis, investigation, resources, data curation, visualization, funding acquisition, writing - original draft and writing - review & editing. **Eiji Yasaka**: methodology, validation, investigation, resources, funding acquisition, writing - original draft and writing - review & editing. **Shinji Kondo**: conceptualization, methodology, validation, formal analysis, investigation, visualization, writing - original draft and writing - review & editing. **Farhana Ferdousi**: conceptualization, methodology, validation, formal analysis, investigation, data curation, visualization, writing - original draft and writing - review & editing. **Mitsutoshi Nakajima**: conceptualization, funding acquisition, project administration, and writing - review & editing. **Hiroko Isoda**: conceptualization, methodology, funding acquisition, project administration, resources, supervision, and writing - review & editing.

All data was generated in-house, and no paper mill was utilized. All authors agree to be accountable for all aspects of the work, ensuring integrity and accuracy.

### Ethics statement

The animal study protocol was approved by the University of Tsukuba Ethics and Animal Welfare Committee (23-328).

### Data availability

Microarray data are deposited to NCBI under accession number GSE293650. Artificial intelligence tools were not employed in this study. Transcriptomic data were analyzed using standard bioinformatics pipelines and statistical packages.

See also APPENDIX A(Tab. 2).

## Supplementary Material

Supplementary information

## Figures and Tables

**Table 1:**
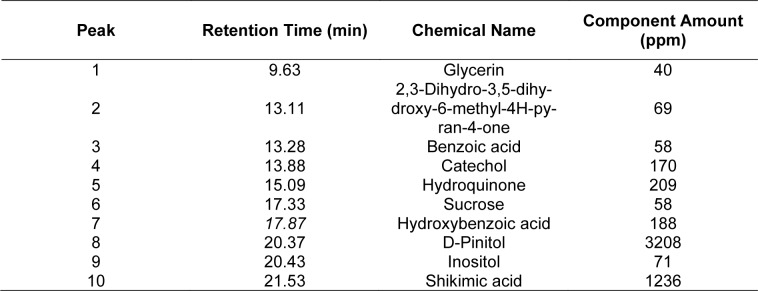
GC/MS Analysis of PN Qualitative and quantitative assessment of 10 peaks identified in PN using GC/MS analysis. Quantitative measurements were standardized with pyrocatechol as the reference.

**Table 2:**
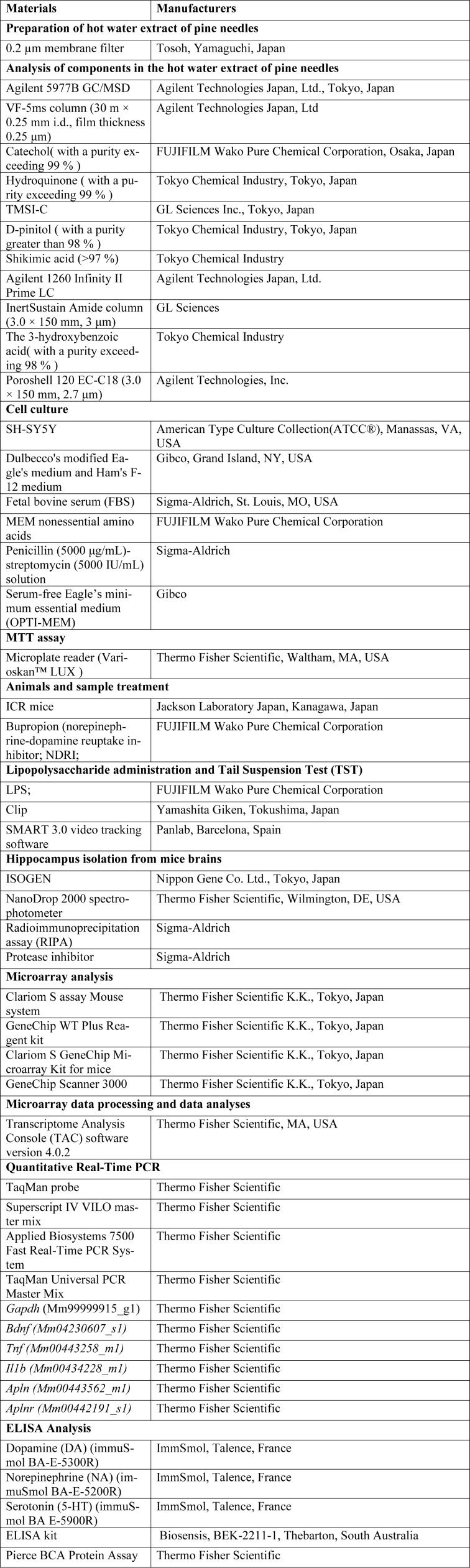
APPENDIX A

**Figure 1:**
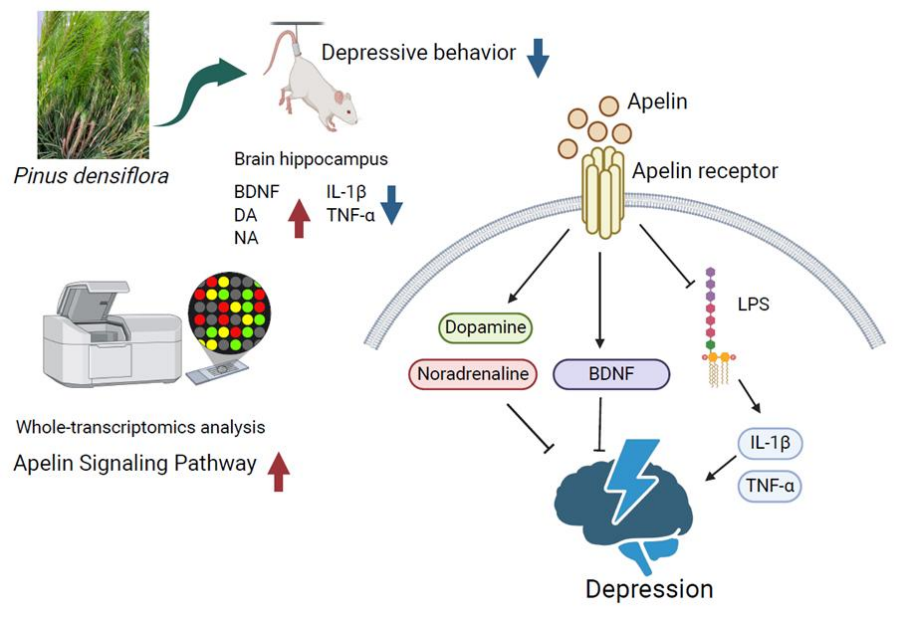
Graphical abstract Antidepressant-like and neuroprotective effects of pine needle extracts: evidence from behavioral, transcriptomic, and biochemical studies
