# Supplementary material for: Antidepressant-like and neuroprotective effects of pine needle extracts: evidence from behavioral, transcriptomic, and biochemical studies
Source: EXCLI J. 2025 Oct 17;24:1372–96. doi: 10.17179/excli2025-8720 (PMC12627992; doi:10.17179/excli2025-8720)
Supplement: Supplementary information [file EXCLI-24-1372-s-001.pdf]

EXCLI Journal 2025;24:1372-1396 – ISSN 1611-2156
Received: July 05, 2025, accepted: September 02, 2025, published: October 17, 2025

Supplementary information to:

Original article:

ANTIDEPRESSANT-LIKE AND NEUROPROTECTIVE EFFECTS OF PINE NEEDLE EXTRACTS: EVIDENCE FROM BEHAVIORAL, TRANSCRIPTOMIC, AND BIOCHEMICAL STUDIES

Hisako Iwahashi Ogawa [1,2], Eiji Yasaka [2], Shinji Kondo [4], Farhana Ferdousi [1,3,4], Mitsutoshi Nakajima [4,5,6], Hiroko Isoda [1,3,4,5,6*]

[1] Degree Programs in Life and Earth Sciences, Graduate School of Science and Technology, University of Tsukuba, Tsukuba, Ibaraki, 305-8572, Japan
[2] Arakawa Chemical Industries, Ltd.,1-3-7, Hiranomachi, Chuo-ku, Osaka,541-0046, Japan
[3] Institute of Life and Environmental Sciences, University of Tsukuba, Tsukuba, Ibaraki, 305-8575, Japan
[4] Alliance for Research on the Mediterranean and North Africa (ARENA), University of Tsukuba, Tsukuba, Ibaraki 305-8572 Japan
[5] Open Innovation Laboratory for Food and Medicinal Resource Engineering (FoodMed-OIL), National Institute of Advanced Industrial Science and Technology (AIST), Tsukuba, Ibaraki, 305-0821, Japan
[6] MED R&D Co. Ltd., Tsukuba, Ibaraki, 305-8572, Japan

* **Corresponding author:** Professor Hiroko Isoda, Institute of Life and Environmental Sciences, University of Tsukuba, Japan1-1-1 Tennodai, Tsukuba City, Ibaraki 305-8572, Japan. E-mail: isoda.hiroko.ga@u.tsukuba.ac.jp

https://dx.doi.org/10.17179/excli2025-8720

This is an Open Access article distributed under the terms of the Creative Commons Attribution License (https://creativecommons.org/licenses/by/4.0/).

EXCLI Journal 2025;24:1372-1396 – ISSN 1611-2156
Received: July 05, 2025, accepted: September 02, 2025, published: October 17, 2025

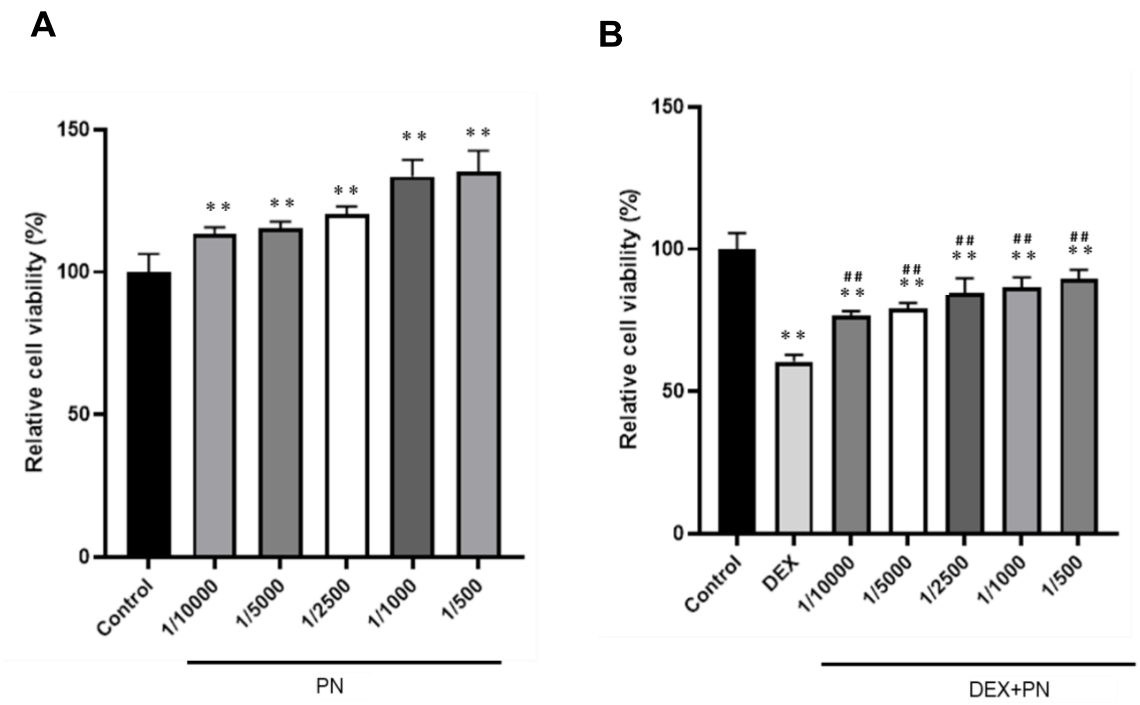

Supplementary Figure 1: Effect of pine needle extract (PN) on **A**) cell viability and **B**) dexamethasone (DEX)-induced changes in human neuroblastoma SH-SY5Y cells. SH-SY5Y cells were treated with PN for 48 h to evaluate cell viability. SH-SY5Y cells were pretreated with PN for 1 h and then treated with 250 µM DEX for 48 h to evaluate neuroprotection. Statistical significance was assessed using one-way ANOVA followed by Tukey's post-hoc test. Each bar represents the mean ± SEM (n = 5 independent experiments). ***p* value < 0.01 vs. control cells, ##*p* value < 0.01 vs. DEX-treated cells.

EXCLI Journal 2025;24:1372-1396 – ISSN 1611-2156
Received: July 05, 2025, accepted: September 02, 2025, published: October 17, 2025

Supplementary Table 1: Detailed heatmap data showing differentially expressed genes (DEGs) categorized into four groups:
・Downregulated in LPS and Upregulated in PN
・Upregulated in LPS and Downregulated in PN
For each group, the table includes the associated Gene Ontology (GO) terms, log p-values (enrichment significance), Enrichment scores, the number of DEGs, and the list of DEGs. This comprehensive dataset provides detailed insights into gene expression patterns underlying the effects of PN and LPS treatment.

		**Downregulated in LPS**				**Upregulated in PN**			
**GO term**	**GO no.**	**LogP**	**Enrich-ment**	**No. of DEGs**	**List of DEGs**	**LogP**	**Enrich-ment**	**No. of DEGs**	**List of DEGs**
fatty acid bio-synthetic pro-cess	GO:0006633	-2.4	3.1	8	ALOX12|ALOX5|CYP1A1|CYP3A4|EDN2|PTGDS|TBXAS1|AKR1C3	-2	2.7	8	ALOX12|GPX4|LTC4S|MIF|PRKAA1|TBXAS1|HACD1|ALOXE3
inflammatory response	GO:0006954	-9	3.1	38	AHSG|ALOX5|BMP2|CD36|CD40LG|CCR3|CCR4|IL1B|IL1RN|IL9|IL13|IDO1|CXCL9|SER-PINA1|PTGDR|PTGER2|RARRES2|S100A8|SELE|SELP|TNF|TNFRSF1A|TNFSF4|CXCR4|AOC3|CD96|NOD1|AGR2|IL36RN|NOX1|CHIA|IL36B|TLR8|UGT1A1|GSDMC|ZC3H12A|H2BC1|NLRP9	-2.6	1.8	26	TMEM258|CASP8|CD36|CD44|S1PR3|ITGAM|MIF|NFK-BIB|PTGER1|RELA|CCL25|SLC11A1|TGFB1|TLR1|MAPKAPK2|LY86|SMPDL3B|PY-CARD|TLR7|PXK|GSDMD|IL1F10|IL17RC|IL33|IL34|NLRP9
cranial nerve development	GO:0021545	-2.6	4.3	6	HOXA1|HOXB1|ATP8B1|SIX1|SLC38A8|TIFAB	-2.3	3.8	6	EPHB1|MAFB|ACKR3|SLI-TRK6|NAV2|DRGX
regulation of epithelial cell differentiation	GO:0030856	-5.3	4.1	15	AQP3|CEA-CAM1|BMP2|FOXE3|GDF2|IL1B|IL13|KRT84|TNF|TNFRSF1A|WNT10B|KRT36|GDF3|ESRP1|OSR1	-2.4	2.6	11	CEA-CAM1|CDH5|CYP27B1|S1PR3|EXTL3|FRZB|NCOA3|KLF7|ATOH8|OSR1|SPRED2
carbohydrate homeostasis	GO:0033500	-2.1	2.3	11	ALOX5|CASR|GCGR|CMKLR2|HK2|FOXA1|LE	-2.1	2.2	12	CASR|FKBP1B|IGFBP5|PRKAA1|RBM4|SRF|TCF7L2|T

EXCLI Journal 2025;24:1372-1396 – ISSN 1611-2156
Received: July 05, 2025, accepted: September 02, 2025, published: October 17, 2025

					P|SER-PINF1|CCN4|SLC12A7|OBP2A				GFB1|KLF7|NR1D1|MBD5|ADGRF5
T cell activa-tion	GO:0042110	-3	2.4	17	CD3E|TNFSF8|CTSL|IFNA1|IFNA5|IL7|IDO1|LEP|MSN|RORC|STAT4|TNFSF4|WNT10B|TNFSF14|TNFSF18|TMEM98|IFNE	-3.1	2.3	19	CASP8|RUNX2|CD4|CD8A|CD44|FKBP1B|IT-PKB|SLC11A1|SPI1|SRF|TGFB1|TP53|TOX|MAFB|CCR9|FOXP1|APBB1IP|ENTPD7|PRR7
glucose home-ostasis	GO:0042593	-2.1	2.3	11	ALOX5|CASR|GCGR|CMKLR2|HK2|FOXA1|LEP|SER-PINF1|CCN4|SLC12A7|OBP2A	-2.1	2.2	12	CASR|FKBP1B|IGFBP5|PRKAA1|RBM4|SRF|TCF7L2|TGFB1|KLF7|NR1D1|MBD5|ADGRF5
innate immune response	GO:0045087	-3.7	2	33	ARG1|C2|DEFA1|CFD|HLA-B|HPX|IFNA1|IFNA5|KLRC2|LGALS3|RARRES2|S100A8|TNF|APOL1|OASL|NOD1|IL36RN|IL36B|TLR8|SLC46A2|CARD9|ULBP1|NEURL3|PGLYRP3|RNF166|APO-BEC3H|SLC30A8|DEFB128|WFDC9|NLRP9|IFNE|LILRA5|DEFB131A	-2.9	1.8	34	SERPING1|CYP27B1|FAU|HLA-B|ITGAM|RPSA|MIF|RELA|RNASE3|SLC11A1|TLR1|TP53|ADAM15|LY86|PQBP1|IFITM2|PLEKHM2|SMPDL3B|PY-CARD|ZDHHC1|TLR7|TRIM68|SPIRE1|GSDMD|RNF39|COLEC12|IL33|TIFA|TRIM6|IL34|DEFB128|STXBP4|NLRP9|STING1
defense re-sponse to Gram-nega-tive bacterium	GO:0050829	-2.1	3.1	7	DEFA1|RARRES2|SELP|NOD1|DEFB128|LCE3B|LCE3C	-2.3	3.1	8	CD4|RNASE3|RPS19|SLC11A1|PY-CARD|GSDMD|TSLP|DEFB128
regulation of cell activation	GO:0050865	-4.5	2.2	31	ALOX12|ARG1|CEA-CAM1|CD3E|CD19|TNFSF8|CD40LG|HLA-DOB|IL1B|IL7|IL13|IDO1|KLRC2|LEP|LGALS3|SELP|SMARCD2|TNF|TNFSF4|WNT10B|TNFSF1	-2.5	1.8	28	ACTA2|ALOX12|CEA-CAM1|CAPN3|CD4|CDKN1A|GP5|IGF1|IT-PKB|MIF|PRKAA1|SPI1|TGFB1|CST7|NR1D1|TOX|RNF4

EXCLI Journal 2025;24:1372-1396 – ISSN 1611-2156
Received: July 05, 2025, accepted: September 02, 2025, published: October 17, 2025

					4|TNFSF18|MMRN1|IL36B|MZB1|BTNL2|SLC46A2|ZC3H12A|PGLYRP3|TIGIT|LILRA5				1|PAXIP1|RABGEF1|PYCARD|SH3KBP1|AMBRA1|VSIR|TSLP|IL33|ZBTB46|PIK3R6|ADGRF5
cellular response to cytokine stimulus	GO:0071345	-3.1	1.9	31	CEACAM1|CXCR5|CCR3|CCR4|CCR8|EDN2|HK2|HPX|IFNA1|IFNA5|IL1B|IL7|IL9|IL13|LEP|CXCL9|ROR2|PRL|STAT4|TFF2|TNF|TNFRSF1A|CXCR4|OASL|TNFSF18|IL36B|RBMX|TEX14|ZC3H12A|IFNE|LILRA5	-2.4	1.7	32	ABCA1|CEACAM1|CD4|CD44|EPHB1|RELA|RPS16|CCL25|SELPLG|SRSF7|SPI1|TP53|NR1D1|ST18|ST3GAL6|IFITM2|CCR9|PTP4A3|FOXP1|RABGEF1|PYCARD|ACKR3|ABCG4|IL1F10|IL17RC|TSLP|IL33|TIFA|TRIM6|IL34|STXBP4|STING1
neuron fate specification	GO:0048665	-2.2	5.4	4	FOXA1|HOXD10|SIX1|TLX3	n/a			
astrocyte development	GO:0014002	-2.1	5.1	4	IL1B|ROR2|S100A8|TNF	n/a			
neurotransmitter receptor internalization	GO:0099590					-2.4	8.8	3	AP2S1|CACNG2|CACNG7
regulation of cell growth	GO:0001558					-3.7	2.3	23	CEACAM1|BMPR2|CDKN1A|CYP27B1|EXTL3|FRZB|IGF1|IGFBP5|IGFBP7|ING1|RYK|SRF|SUPV3L1|TGFB1|TP53|URI1|ADAM15|CEP43|FAM107A|TWF2|FOXP1|CACNG7|RTN4R
regulation of nervous system development	GO:0051960					-3	2.1	23	BMPR2|EPHB1|MBD1|RELA|RYK|SRF|TGFB1|TIAM1|TP53|CST7|NR1D1|TWF2|TRAK1|PARD3|LRRN1|CTDSP1|

EXCLI Journal 2025;24:1372-1396 – ISSN 1611-2156
Received: July 05, 2025, accepted: September 02, 2025, published: October 17, 2025

									CLSTN2|RTN4R|SLI-TRK6|NKX6-2|IL33|IL34|LINGO2
dendrite morphogenesis	GO:0048813					-3	4.4	7	EPHB1|MEF2A|KLF7|WASL|ABI1|TRAK1|SHANK1
nerve development	GO:0021675					-2.5	3.3	8	EPHB1|GABRA5|NTF3|MAFB|ACKR3|SLI-TRK6|NAV2|DRGX
regulation of glial cell differentiation	GO:0045685					-2.5	3.6	7	MBD1|RELA|TGFB1|NR1D1|NKX6-2|IL33|IL34
glial cell proliferation	GO:0014009					-2.3	5.6	4	NFIA|TP53|IL33|IL34
brain development	GO:0007420					-2.2	1.6	30	APAF1|COL3A1|COX6B1|EMX2|EPHB1|GPX4|GRIN2C|ITGAM|PBX3|RFX4|RYK|ATXN1|STIL|SRF|SSTR2|TP53|CDK5R2|TOX|MAFB|TACC3|NCOA6|LHX6|FOXP1|CEND1|MEIS3|SHROOM4|RTN4R|FAT4|DNAJC30|SLC32A1
regulation of gliogenesis	GO:0014013					-2.2	2.9	8	MBD1|RELA|TGFB1|TP53|NR1D1|NKX6-2|IL33|IL34

		Upregulated in LPS				Downregulated in PN			
**GO term**	**GO no.**	**LogP**	**Enrich-ment**	**No. of DEGs**	**List of DEGs**	**LogP**	**En-rich-ment**	**No. of DEGs**	**List of DEGs**
response to virus	GO:0009615	-2.7	2.1	19	ADAR|DEFA1|DEFA3|GSDME|HSP90AA1|IFI16|STMN1|MBL2|NCBP1|XPR1|TBKBP1|NT5C3A|POLR3K|USP29|ZC3H12A|ZBP1|TRIM56|UNC13D|SERINC5	-4.6	2.4	28	ADAR|ADARB1|ARF1|BCL2L1|BNIP3L|CALR|AP1S1|DDX1|DDX3X|EEF1G|EIF5A|FMR1|HIF1A|HSP90AA1|RPSA|STMN1|PCBP2|TPT1|DCLK1|XPR1|RB1CC1|TOMM70|ZMYND11|HNRN-PUL1|LSM14A|GPAM|UBL7|DHX36
regulation of cell cycle process	GO:0010564	-2.6	1.7	31	BID|CXCR5|CDC42|CENPE|DDB1|ECT2|FHL1|HSPA1A|MKI67|TIME-LESS|TAOK2|ROCK2|ADAMTS1|KIF20B|TTI1|KLHL21|BTN2A2|GIPC1|TPX2|OR1A2|NUSAP1|SPDL1|RIOK2|NCAPG|CEP97|ATAD5|CDT1|DOT1L|WDR62|CENATAC|ZC3H12D	-4	1.8	43	AKT1|APBB2|APC|ATRX|BCL2L1|CALM1|CALR|CCNG1|CCNH|CDC42|CLTC|DCTN1|DDX3X|DYNC1H1|EIF4G1|H2AX|HNRNPU|CDK16|CDK17|CDK14|PTEN|RAD21|RDX|UBE2E2|ZNF207|RAE1|RAB11A|BUB3|TAOK2|PSME3|PLK2|ZWINT|FAM107A|PLCB1|ATXN10|ANKRD17|BA-BAM1|FBXW7|AM-BRA1|MTA3|WNK1|MAP9|PPP1R9B
carbohydrate cat-abolic process	GO:0016052	-2.6	3.1	9	CHIT1|FUT1|MPI|PGK2|TKTL1|NEU3|NUDT5|OG-DHL|GLB1L3	-2.3	2.7	10	ALDOC|HEXB|HK1|OGDH|PGK1|PKM|RB1CC1|GABARAPL1|WIPI2|ATG2B
positive regulation of defense re-sponse	GO:0031349	-3.9	2.3	24	KLK3|FABP4|HSPA1A|HSP90AA1|IFI16|IRAK2|KLRC1|MBL2|MEFV|NPAS2|PAK3|UCN|IL1RL1|PJA2|HEXIM1|CD226|CD160|STAP1|USP29|ZBP1|TRIM56|OTULIN|NLRP12|REG3G	-2	1.7	23	CD47|CD81|CNR1|CREBBP|CTSS|DDX3X|GRN|HMGB1|HSP90AA1|HSPD1|LAMP1|MEF2C|PAK1|CX3CL1|SFPQ|PLA2G7|MATR3|PJA2|TOMM70|ANKRD17|LSM14A|AKIRIN2|RTN4

regulation of actin cytoskeleton organization	GO:0032956	-6.1	3.1	25	CDC42|CCN2|ECT2|STMN1|MET|MYOC|NEB|PAK3|RGS4|CCL21|TPM1|TAOK2|ROCK2|AVIL|CDC42EP4|RND1|SNX9|MAGEL2|KIRREL1|CARMIL1|LMOD3|CRACD|XIRP2|MTPN|ARPIN	-6.9	2.9	31	ADD2|ADD3|ARF1|CAPZA2|CD47|CDC42|FLNA|MTOR|GPM6B|STMN1|PAK1|PFN1|PFN2|PIK3CA|RDX|CX3CL1|SPTAN1|SPTBN1|TPM1|CDK5R1|TAOK2|ARPC3|ARPC2|ABI2|CORO2B|NCKAP1|FAM107A|CYFIP1|CYFIP2|STAU2|MTPN
regulation of stress fiber assembly	GO:0051492	-2.8	3.7	8	CDC42|CCN2|STMN1|MET|MYOC|TPM1|ROCK2|CARMIL1	-2.6	3.1	9	CD47|CDC42|MTOR|STMN1|PAK1|PFN1|PFN2|TPM1|CORO2B
cytoskeleton-dependent cytokinesis	GO:0061640	-2.8	3.3	9	ECT2|STMN1|MTMR4|ROCK2|EXOC5|ZNF365|KIF4A|NUSAP1|SNX9	-3.1	3.1	11	ANK3|APC|ARF1|STMN1|ORC4|SPTBN1|ACTR3|ZNF365|SPIRE1|EXOC4|MAP9
negative regulation of intracellular signal transduction	GO:1902532	-2.7	1.8	30	BID|ESR1|GSTP1|HELLS|HSPA1A|IFI16|IGBP1|ISL1|STMN1|MEFV|MET|MMP3|MYOC|SERPINB3|VDAC2|RANBP9|BTN2A2|HYOU1|PRAME|TXNDC12|INSIG2|RRN3|NLRP2|YJU2|MAPKAP1|ATAD5|ZC3H12A|WDR24|NLRP12|ADGRG3	-8.7	2.4	54	AKT1|APOE|BCL2L1|CALR|CRY2|CSNK1A1|DDX3X|DUSP8|EPHA4|MTOR|HIF1A|HSPA5|ITPR1|STMN1|MARK3|NDUFC2|CNOT2|CCN3|PAFAH1B1|PCBP2|SERPINE2|PPIA|PPM1A|PPM1B|PPP2CB|PPP6C|PRKACA|PRKACB|PRKAR1A|PRKAR1B|MAP2K5|PTEN|SKI|SOD2|TMBIM6|TPT1|UBE2D1|UCHL1|VCP|VDAC2|YWHAZ|SYNGAP1|AKT3|ZMYND11|SIRT3|STRN3|NLK|FBXW7|NPLOC4|ASH1L|LMO3|SAR1A|ARHGAP12|SPRED2
microtubule cytoskeleton organization involved in mitosis	GO:1902850	-4.2	3.9	12	CENPE|KPNB1|STMN1|PTPA|TPX2|FBXW11|KIF4A|NUSAP1|SPDL1|MAP1S|WDR62|MZT1	-4.2	3.4	14	DCTN1|DYNC1H1|FLNA|GJA1|STMN1|MAP4|PAFAH1B1|VCP|ZNF207|RAB11A|NUDC|PLK2|LSM14A|MAP9

EXCLI Journal 2025;24:1372-1396 – ISSN 1611-2156
Received: July 05, 2025, accepted: September 02, 2025, published: October 17, 2025

negative regulation of response to oxidative stress	GO:1902883	-2.6	11	3	MEAK7|MCTP1|NCOA7	-2.3	8.3	3	GGT7|USP25|OXR1
chemokine binding	GO:0019956	-2.2	5.3	4	CXCR5|CCR6|CCR8|ZFP36	n/a			
cytokine receptor activity	GO:0004896	-2.2	3.2	7	CXCR5|CCR6|CCR8|F3|GFRA1|IL1RL1|IL22RA2	n/a			
cellular response to cytokine stimulus	GO:0071345					-3.8	1.8	43	AKT1|SLC25A5|ATP5F1B|CALM1|CD47|CDC42|EIF5A|EPHA4|EPHA7|FLNB|PDIA3|HIF1A|HNRNPU|HSPA5|HSP90AB1|HSPD1|KIT|LIFR|MAT2A|NFYB|NTRK2|NTRK3|PRKACA|MAPK1|MAP2K7|RAF1|CX3CL1|TLE4|UGCG|ADAM23|INA|EEF1E1|SPOCK2|ACTR3|MRAS|PLCB1|WBP1L|ABCG4|WNK1|ARID5B|VPS26B|EPHA6|SLC27A1
positive regulation of dendritic cell cytokine production	GO:0002732					-2.4	9.1	3	DDX1|KIT|DHX36
positive regulation of myeloid leukocyte cytokine production involved in immune response	GO:0061081					-2.4	4.7	5	DDX1|KIT|NR4A3|RTN4|DHX36
positive regulation of cytokine production	GO:0001819					-2.4	1.7	27	B2M|BSG|CD81|DDX1|DDX3X|HDAC2|HIF1A|HK1|HMGB1|HSP90AA1|HSPD1|KIT|MAPK9|RAB1A|RAD21|CX3CL1|SORL1|SPTBN1|NR4A3|TOMM70|AGPAT1|PL

EXCLI Journal 2025;24:1372-1396 – ISSN 1611-2156
Received: July 05, 2025, accepted: September 02, 2025, published: October 17, 2025

									CB1|AKI-RIN2|SULF2|RTN4|DHX36|SCAMP5
positive regulation of programmed cell death	GO:0043068					-4.7	2.1	37	APBB2|APC|BCL2L1|BNIP3L|CDC42|CNR1|DDX3X|EIF5A|EPHA7|GRN|GRIN2A|PDIA3|HMGB1|HSPD1|IGF2R|ITPR1|KCNMA1|MAL|MEF2C|MAPK9|RPS6|SFPQ|SOD2|SP1|SRPK2|TIA1|VDAC1|NCOA1|TSC22D1|CDK5R1|MAGED1|EEF1E1|RBM5|OLFM1|ARL6IP5|FBXW7|CSRNP3
regulation of neuron apoptotic process	GO:0043523					-9	3.8	29	BCL2L1|CDC42|EPHA4|EPHA7|GRN|GRIK2|HIF1A|MEF2C|NTRK2|PIK3CA|PRKCG|MAP2K7|PTPRZ1|RAD21|CX3CL1|SET|SOD2|SRPK2|SYN-GAP1|CDK5R1|SIG-MAR1|CHL1|FAIM2|HIPK2|OXR1|FBXW7|AM-BRA1|CPEB4|AGAP2
apoptotic mitochondrial changes	GO:0008637					-3.9	4.6	9	AKT1|SLC25A4|BCL2L1|BNIP3L|HSPD1|PPP2CB|SOD2|VDAC2|VPS35
apoptotic signaling pathway	GO:0097190					-3.7	2.3	23	BCL2L1|BNIP3L|DDX3X|PDIA3|ITPR1|PDK2|PRKCA|MAPK9|RAF1|CX3CL1|SOD2|TMBIM6|VDAC2|BAG6|CUL1|RB1CC1|RNF41|ARL6IP5|FASTK|FAIM2|HIPK2|CYCS|HIPK1

EXCLI Journal 2025;24:1372-1396 – ISSN 1611-2156
Received: July 05, 2025, accepted: September 02, 2025, published: October 17, 2025

Supplementary Table 2: Top 20 Genes Upregulated by PN Treatment
The top 20 genes upregulated in the hippocampus of mice following oral administration of PN in the TST model. Genes are ranked according to fold-change values obtained through microarray analysis.

Gene Symbol	Description	Fold Change	*p* Value	Biological Functions
Wipf2	WAS/WASL interacting protein family, member 2	4.57	0.0003	actin binding<br>cytoplasm
Retsat	retinol saturase (all trans retinol 13,14 reductase)	4.00	0.0047	oxidoreductase activity<br>lipid metabolic process
Rpp25	ribonuclease P/MRP 25 subunit	3.84	0.0002	nucleic acid binding<br>rRNA processing
Spi1	spleen focus forming virus (SFFV) proviral integration oncogene	3.61	0.0004	positive regulation of myeloid dendritic cell chemotaxis, lipopolysaccharide-mediated signaling pathway
Ddx26b	DEAD/H (Asp-Glu-Ala-Asp/His) box polypeptide 26B	3.30	0.0008	snRNA 3'-end processing
Rexo1	REX1, RNA exonuclease 1 homolog (S. cerevisiae)	3.07	0.0004	nucleic acid binding<br>hydrolase activity
Ctdsp1	CTD (carboxy-terminal domain,<br>RNA polymerase II, polypeptide A) small phosphatase 1	3.01	0.0001	phosphoprotein phosphatase activity<br>myosin phosphatase activity
Mid1	midline 1	2.81	0.0151	positive regulation of stress-activated MAPK cascade
Grina	glutamate receptor, ionotropic,<br>N-methyl D-aspartate-associated protein 1 (glutamate binding)	2.77	0.0021	reticulum stress-induced intrinsic apoptotic signaling pathway<br>transmembrane transporter binding
Rpsa	ribosomal protein SA	2.70	0.0075	synapse<br>neuronal cell body
Fcrls	Fc receptor-like S, scavenger receptor	2.66	0.0111	transmembrane signaling receptor activity
Rps16	ribosomal protein S16	2.65	0.0038	translation at presynapse, translation at postsynapse

EXCLI Journal 2025;24:1372-1396 – ISSN 1611-2156
Received: July 05, 2025, accepted: September 02, 2025, published: October 17, 2025

Ppp1ca	protein phosphatase 1, catalytic subunit, alpha isoform	2.63	0.0167	translation at presynapse, translation at postsynapse
Psmd13	proteasome (prosome, macropain) 26S subunit, non-ATPase, 13	2.62	0.0004	ubiquitin-dependent protein catabolic process
Tmem258	transmembrane protein 258	2.60	0.0028	inflammatory response, epithelial cell apoptotic process
Prr14	proline rich 14	2.57	0.0001	muscle organ development
Rpl23a	ribosomal protein L23A; small nucleolar RNA, C/D box 42B	2.54	0.0359	translation at presynapse, translation at postsynapse
Lrp11	low density lipoprotein receptor-related protein 11	2.52	0.0434	multicellular organismal response to stress, response to immobilization stress
Igfbp7	insulin-like growth factor binding protein 7	2.52	0.0004	insulin-like growth factor binding, growth factor binding
Ndufb9	NADH dehydrogenase (ubiquinone) 1 beta subcomplex, 9	2.40	0.0039	proton motive force-driven mitochondrial ATP synthesis

Supplementary Table 3: Top 20 Genes Downregulated by PN Treatment
The top 20 genes downregulated in the hippocampus of mice following oral administration of PN in the TST model. Genes are ranked according to fold-change values obtained through microarray analysis.

Gene Symbol	Description	Fold Change	*p* Value	Biological Functions
Kcnj16	potassium inwardly-rectify-ing channel, subfamily J, member 16	-5.72	0.0057	inward rectifier potassium channel activity monoatomic ion transport
Zic1	zinc finger protein of the cerebellum 1	-4.2	0.0122	nervous system develop-ment,hippocampus devel-opment, RNA polymerase II transcription regulatory region sequence-specific DNA binding
Nt5dc3	5-nucleotidase domain containing 3; 5'-nucleo-tidase domain containing 3	-2.67	0.0012	5'-nucleotidase activity mitochondrion
Ano2	anoctamin 2	-2.58	0.0262	intracellular calcium acti-vated chloride channel ac-tivity, neuron projection
Hells	helicase, lymphoid specific	-2.57	0.0046	ATP binding
Glo1	glyoxalase 1	-2.47	0.0128	GPCR, family 3, extracel-lular calcium-sensing re-ceptor-related
Fstl1	follistatin-like 1	-2.38	0.0017	lactoylglutathione lyase activity
Zfp729a	zinc finger protein 729a	-2.19	0.0086	pheromone receptor activ-ity
Xlr4b	X-linked lymphocyte-regu-lated 4B	-2.18	0.0118	dopamine secretion positive regulation of syn-apse assembly
Isl1	ISL1 transcription factor, LIM/homeodomain	-2.12	0.0009	negative regulation of neuron differentiation negative regulation of in-flammatory response
Slc25a31	solute carrier family 25 (mitochondrial carrier; ad-enine nucleotide transloca-tor), member 31	-2.03	0.0362	cell differentiation, mito-chondrial ATP transmem-brane transport
Thoc2	THO complex 2	-2.00	0.0099	negative regulation of neuron projection devel-opment

EXCLI Journal 2025;24:1372-1396 – ISSN 1611-2156
Received: July 05, 2025, accepted: September 02, 2025, published: October 17, 2025

Gstp1	glutathione S-transferase, pi 1	-1.98	0.0002	regulation of stress-activated MAPK cascade, inflammatory response
Cdh19	cadherin 19, type 2	-1.98	0.0128	cell migration, cell-cell adhesion
Rasd1	RAS, dexamethasone-induced 1	-1.98	0.0158	negative regulation of DNA-templated transcription
Lrrc16a	leucine rich repeat containing 16A	-1.98	0.0264	positive regulation of stress fiber assembly
Slc34a1	solute carrier family 34 (sodium phosphate), member 1	-1.98	0.0099	monoatomic ion transport
Cps1	carbamoyl-phosphate synthetase 1	-1.96	0.0364	ATP binding, potassium ion binding
Tbkbp1	TBK1 binding protein 1	-1.94	0.0352	protein binding, zinc ion binding
Cd19	CD19 antigen	-1.93	0.0132	B cell proliferation, regulation of B cell receptor signaling pathway
